# “Quality of prenatal and maternal care: bridging the know-do gap” (QUALMAT study): an electronic clinical decision support system for rural Sub-Saharan Africa

**DOI:** 10.1186/1472-6947-13-44

**Published:** 2013-04-10

**Authors:** Antje Blank, Helen Prytherch, Jens Kaltschmidt, Andreas Krings, Felix Sukums, Nathan Mensah, Alphonse Zakane, Svetla Loukanova, Lars L Gustafsson, Rainer Sauerborn, Walter E Haefeli

**Affiliations:** 1 Department of Clinical Pharmacology and Pharmacoepidemiology, Medizinische Klinik (Krehl Klinik), University Hospital of Heidelberg, Im Neuenheimer Feld 410, Heidelberg, D - 69120, Germany; 2 Department of Public Health, University of Heidelberg, Im Neuenheimer Feld 327, Heidelberg, D - 69120, Germany; 3 Muhimbili University of Health and Allied Sciences (MUHAS), Directorate of Information and Communication Technology, P.O. Box 65001, Dar Es Salaam, TZ, Tanzania; 4 Navrongo Health Research Centre, P.O. Box 114, Navrongo, GH, Ghana; 5 Centre de Recherche en Santé de Nouna (CRSN), Nouna, BF, BP 02, Burkina Faso; 6 Department of Laboratory Medicine (LABMED), Division of Clinical Pharmacology (C1:68), Karolinska Institutet, Karolinska University Hospital, Stockholm, SE-141 86, Sweden

**Keywords:** Guideline adherence, Clinical decision support systems, Medical informatics, Maternal health services, Pregnancy, Prenatal care, Perinatal care, Millennium development goal, Motivation by information technology, Rural maternal healthcare

## Abstract

**Background:**

Despite strong efforts to improve maternal care, its quality remains deficient in many countries of Sub-Saharan Africa as persistently high maternal mortality rates testify. The QUALMAT study seeks to improve the performance and motivation of rural health workers and ultimately quality of primary maternal health care services in three African countries Burkina Faso, Ghana, and Tanzania. One major intervention is the introduction of a computerized Clinical Decision Support System (CDSS) for rural primary health care centers to be used by health care workers of different educational levels.

**Methods:**

A stand-alone, java-based software, able to run on any standard hardware, was developed based on assessment of the health care situation in the involved countries. The software scope was defined and the final software was programmed under consideration of test experiences. Knowledge for the decision support derived from the World Health Organization (WHO) guideline “Pregnancy, Childbirth, Postpartum and Newborn Care; A Guide for Essential Practice”.

**Results:**

The QUALMAT CDSS provides computerized guidance and clinical decision support for antenatal care, and care during delivery and up to 24 hours post delivery. The decision support is based on WHO guidelines and designed using three principles: (1) Guidance through routine actions in maternal and perinatal care, (2) integration of clinical data to detect situations of concern by algorithms, and (3) electronic tracking of peri- and postnatal activities. In addition, the tool facilitates patient management and is a source of training material. The implementation of the software, which is embedded in a set of interventions comprising the QUALMAT study, is subject to various research projects assessing and quantifying the impact of the CDSS on quality of care, the motivation of health care staff (users) and its health economic aspects. The software will also be assessed for its usability and acceptance, as well as for its influence on workflows in the rural setting of primary health care in the three countries involved.

**Conclusion:**

The development and implementation of a CDSS in rural primary health care centres presents challenges, which may be overcome with careful planning and involvement of future users at an early stage. A tailored software with stable functionality should offer perspectives to improve maternal care in resource-poor settings.

**Trial registration:**

http://www.clinicaltrials.gov/NCT01409824.

## Introduction

In 2010, a tragic 287,000 maternal deaths are estimated to have taken place worldwide. The countries in sub-Saharan Africa (SSA) carried the largest burden of these maternal deaths (56%), corresponding to an average maternal mortality ratio (MMR) of 500 deaths per 100,000 live births and a life time risk of 1 in 39 for a woman to die of maternal causes [1]. In 2010 the MMR was estimated to be 460 per 100,000 in Tanzania, 300 in Burkina Faso, and 350 in Ghana [1]. The range of these figures has been confirmed by others [2]. Despite an overall decline of MMR between 1990-2010 of 41% for SSA, the achievement of the Millennium Development Goal No. 5, which requests a 75% reduction of the MMR between 1990 and 2015, remains a tremendous challenge [1]. In addition 2.9 million babies died in 2011 in the first four weeks of life, most of them in developing countries. In SSA this translates into 33 deaths per 1000 newborns. While the rate for under-five deaths is declining the neonatal death rate is increasing, suggesting that measures around delivery may be of particular importance to influence this rising neonatal mortality rate [3].

The availability of skilled and motivated providers is central to progress in maternal care [4-6]. Unfortunately studies have shown that even well trained health staff frequently do not perform to the best of their ability [7-9], and that differences can be observed between how health providers know a task should be performed and how they actually perform it. This so-called “know-do gap” [10] is particularly critical in maternal care where providers need to be proactive, observe warning signs closely, and take appropriate actions rapidly and in anticipation of events. Delayed or incorrect decisions may cause either the loss of the mother’s or the baby’s life, or may cause permanent disability resulting in long- term consequences for an entire family.

A major cause of the know-do gap is a health worker’s level of motivation [11]. Motivation at work holds the key to performance of individuals and organizations alike [12]. It would seem fair to assume that highly motivated workers with suboptimal competence and skills will not perform well and, conversely, that low motivation may limit the performance of even those health workers that command optimal levels of competence. In addition, other factors such as the culture of a professional environment or the openness for learning, also influence performance.

In an ideal world, knowledge is correctly applied by motivated health workers in an environment of appreciation and professional exchange. Interventions to improve clinical performance promise better chances of success if they improve health worker competence and motivation, as well as the work environment.

Today, health care information technology (HIT) provides tools that facilitate the application of knowledge at the point of care and thereby simplify adherence to guidelines, which can improve practitioner’s performance, and ultimately patient care. These promising effects have been confirmed in developed countries [13,14]. Meanwhile, resource-poor countries have started to explore and apply the opportunities of HIT systems [15] and consider this as one of the key areas of development activities presently. Amongst other benefits, HIT systems are seen to hold the potential for such countries to leap-frog technological steps of development [16]. However, improvements of this nature can only materialize if HIT systems are customized to the needs of local practitioners and take the challenging environment of resource-poor countries into consideration. Attention needs to be paid to the specifics of the user population, environmental demands, and implementation challenges in rural areas with unreliable access to electricity, technicians, and more highly trained cadres of health workers. In addition, systems have to be thoroughly tested prior to their widespread application as experiences in the developed world have shown that HIT systems may also have unforeseen detrimental effects on patient care [14,15].

If HIT systems are thoroughly developed and piloted they hold the potential to support the competences of local health workers by providing easy access to learning tools, or even information exchange to local and distant colleagues, and possibilities for continuous updates of treatment guidelines. Computerized decision support may even catalyze decision-making in situations where there is time pressure and no possibility to seek advice from other professional colleagues. Indeed, such support may reassure health staff working in professional isolation and serve to improve the work environment, enhance motivation, and increase the experience of competency. The very use of Information Technology (IT) can be fun and motivating in itself [17] and using a well designed HIT, whilst experiencing increased competency, should improve clinical performance.

“Quality of prenatal and maternal care: Bridging the know-do gap (QUALMAT)” is a research project funded as part of the 7th Framework Programme of the European Union (grant agreement 22982). It is a collaboration between the Centre de Recherche en Santé de Nouna (Burkina Faso), Ghent University (Belgium), Heidelberg University (Germany), Karolinska Institutet (Sweden), Muhimbili University of Health and Allied Sciences (Tanzania), and Navrongo Health Research Centre (Ghana). The study seeks to improve the quality of such care at primary facility level in Ghana, Burkina Faso, and Tanzania. The study works on the assumption that health provider’s competence and motivation interact with the work environment to give rise to the work effort that produces clinical performance [18,19]. One of the main aims of this study was to achieve the development of a clinical decision support system (CDSS) for pregnancy care in resource-poor environments and to analyse its potential to positively impact upon the competence and motivation of maternal care providers as well as their work environment, thus improving the quality of care.

The objective of this paper is to describe the QUALMAT study and the process of the development of the QUALMAT CDSS prior to its use. A second intervention within the project is the introduction of performance-based incentives, the details of which will be reported elsewhere. Observation of the use of the CDSS in the given environment will later allow conclusions to be drawn regarding the usefulness of such tools for health care provision in rural, resource-poor settings.

## Methods

### The QUALMAT study settings

**Figure 1:**
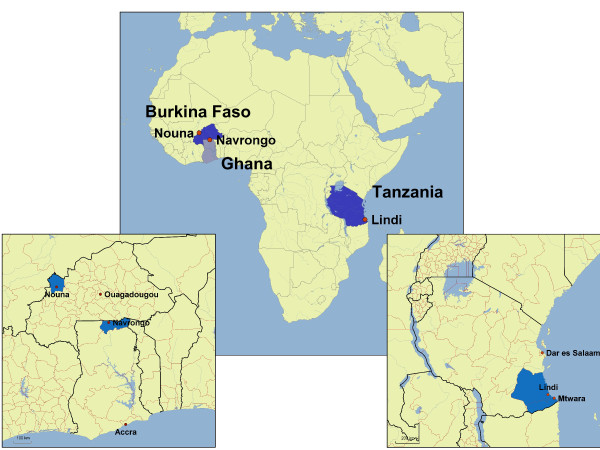
Countries and specific study districts participating in the QUALMAT project.

The CDSS is part of a complex intervention plan within the QUALMAT study. The development of the CDSS preceded the interventions at the study sites. Study activities of the project are carried out at rural primary health care centres in the three countries in SSA (Figure 1). In each country both intervention and control districts were chosen according to several criteria. The study districts were particularly disadvantaged based on comparisons within the countries. Study sites are public facilities at least 10 km away from a town and with a minimum of infrastructure. Availability of electricity was not a prerequisite and was organized through the project where necessary. All the health centres involved in the study have maternity facilities equipped to accommodate uncomplicated deliveries including a 24-hour observation period after delivery. However, none of the sites is equipped or staffed to provide full emergency obstetric care, for example with assisted vaginal delivery methods. Staff members at the sites are health professionals with 1-3 years of training, but no physicians are present. All health centres included in the study are no more than 2 hours drive from a district hospital where patients can be referred. Ambulances from the district health authorities are available upon request (Ghana and Burkina Faso, partly Tanzania) or ambulances are part of the facilities (most facilities in Tanzania). The local district hospitals provide full emergency obstetric care including the possibility for caesarean sections. One district with 6 study sites in each country will be subject to the intervention package, whereas the control district will have no interventions. In Ghana the intervention district is Kassena – Nankana and the control sites are located in Builsa. In Burkina Faso the intervention district is Nouna and the control sites are in Solenzo. In Tanzania, the intervention district is Lindi Rural and the control sites are situated in Mtwara Rural.

The QUALMAT project consortium, in close collaboration with local health authorities at national and sub-national level, has planned to introduce a CDSS and a performance-based incentive scheme in the research sites of the intervention districts. The development and introduction of these interventions is accompanied by research projects, whereby the quality of maternal care, the competence and motivation of those providing such care, and the cost of the interventions is assessed before, during, and after their introduction. In addition, specific studies will be conducted to evaluate the CDSS to analyse adoption and usability of the CDSS, attitudes towards computer use, and the influence of the decision support on medical care and on the workflow at the healthcare centres

Ethical clearance for the QUALMAT study was provided by the Ethics Committee of the Medical Faculty, University of Heidelberg, Germany (ref. S-173/2008), the Institutional Review Board of the Navrongo Health Research Centre, Ghana (ref. NHRCIRB 085), the Muhimbili University of Health and Allied Sciences Ethical Review Committee, Tanzania (ref. MU/RP/AEC/Vol.XIII/96) and the Ethics Committee for Health Research, Burkina Faso (ref. 2010.05/CLE/CRSN).

### QUALMAT CDSS development

The development of the QUALMAT CDSS tried to anticipate critical factors for success of clinical decision support as identified by Kawamoto and colleagues [14]. Future users, medical experts, and IT specialists jointly developed the CDSS using the following steps:

1. Assessment of needs, definition of software scope, and assessment of software interfaces

2. Programming of pilot software with iterations after discussion with future user

3. Programming of the final software and 3 iterative test phases aimed to ensure high quality of the final software (2 software release candidates with several versions)

4. Final implementation

During initial visits to study sites, the local situation of the rural health care facilities in all three countries and the workflow of patient care at the sites were assessed. Future users - mostly, as expected, computer illiterates or individuals with minimal computer experience - and local representatives of the health system from both national and sub-national levels described their environment and outlined their expectations.

There had been no previous HIT projects in the targeted districts for interventions with the exception of Ghana, where the expansion of the national health insurance scheme included the introduction of computers for administrative work at the faculties. In general, HIT projects previously known to the project partners in SSA were mainly focused on facilitating administrative work. Therefore the concept of a CDSS initially required detailed discussions to reach a common understanding regarding the conceptual possibilities of decision support at the point of care. In a second step, about 15 months into the project, a pilot software showing possible functionalities and options was programmed and representatives from all levels of the health system, including future users as stakeholders of the project were asked for their feedback in personal meetings. This was done after future users and individuals from district health authorities had had the opportunity to experiment with using the pilot software. In addition, local research partners investigated the prototype in detail. Feedback on functionality, style, and administrative necessities of the system were received and directed the development phase of the software. At that stage the future users and district health authorities were by now familiar with the concept of decision support, and were very active in expressing their expectations and in proposing changes and additional functionality. The development team was well briefed for the final programming and a first release candidate of the final version was rolled out after an additional 6 months. This release candidate underwent testing and was translated into local languages before being forwarded to the study sites 3 months later.

**Figure 2:**
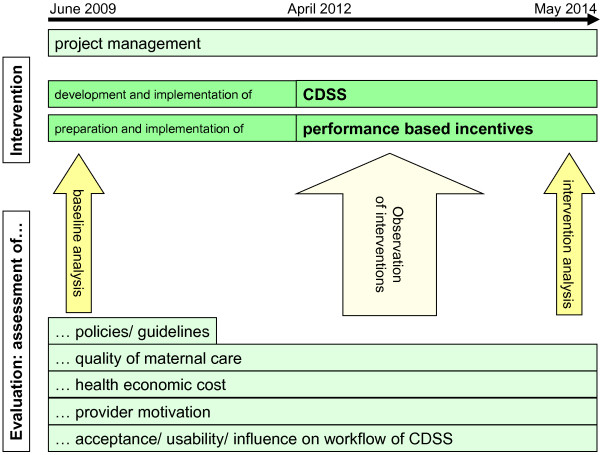
The QUALMAT study: QUALMAT interventions, including the CDSS and accompanying studies.

The test phase for the release candidates included a first part, where the system was tested with use cases and mock patients and a second iteration, where real patient data were entered retrospectively, without using the system for patient care. Care was taken, that a thorough test phase with all pre-planned steps was completed even when it became obvious that timelines for the actual start of the implementation were delayed. A repeated test phase with the second release candidate was added for a final feedback on all changes prior to implementing the final version. The detection of possible problems with the software before its use for patient care was given highest priority. Translation of the system proved to be challenging, whilst conducting the needed training sessions and feedback rounds took a great deal of effort on the part of the local research team. This too was considered essential to ensure that the software could be successfully introduced in the routine care at the study sites. The 3 iterations of testing release candidate versions with iterative programming to accommodate comments received from future users and the task of translations took an unexpected 12 months. Taking account of all the steps - as Figure 2 shows - the initial planning and assessment, programming and testing the pilot versions and programming and testing the release candidate versions - the final version was ready to be implemented for patient care about 3 years after the QUALMAT study consortium had met for the first time.

#### Decision support

The decision support is designed to support routine antenatal maternal care and the delivery - from the woman’s arrival at the facility until she is discharged – which should not happen earlier than 24 hours after delivery. The scope of the project did not allow for the inclusion of postnatal care beyond the post-delivery observation. The knowledge for the decision support was derived from the WHO guidelines “Pregnancy, Childbirth, Postpartum and Newborn Care; A Guide for Essential Practice” [20]. This guideline was compared to the local guidelines of the intervention countries [21]. Differences between the two sources were discussed with local medical experts available within the research partners groups, and minor adaptations were made as per their suggestions.

## Results

The CDSS is a stand-alone application in Java 6 (Oracle America Inc., Redwood Shores, California, USA, freeware). Minimum technical hardware requirements for the software are 1 GB random access memory, Java software, which is platform independent, and a monitor with a resolution of at least 1024×768. Prerequisites were kept to a minimum to allow for broad application and dissemination in the future. The system was developed in English for use in Ghana and later translated into French for use in Burkina Faso, and Kiswahili for use in Tanzania. Translation was a major task therefore an additional translation tool was developed which would also facilitate translation into further languages of future interest.

### Structure of user interface and contents

The software consists of 4 parts:

(1) User interface

(2) Data base for patient data (xml)

(3) Algorithms to screen entered values in the database

(4) Training and information documents

The user interface allows for the system to be operated with only minimal computer knowledge. The interface supports and proposes complete actions for antenatal care and facilitates the supervision of patients under delivery. Users are able to enter and review patient data, which will be stored in an xml database. The xml data may be used for data analysis or other project needs. Algorithms derived from the above mentioned guidelines screen values in the xml database.

In addition, documents may be added to the software at various locations, for example for training purposes. A set of international documents (mostly WHO) has been centrally included and local research teams have added documents from their national or district health authorities.

### Decision support for maternal and perinatal care

The decision support has three different approaches, identified as likely useful in rural health care environments:

(1) Guidance through routine actions in maternal and perinatal care

(2) Integration of clinical data to detect situations of concern by algorithms based on guidelines

(3) Electronic partograph for observation of the progress of delivery up to 24 hours post delivery

**Figure 3:**
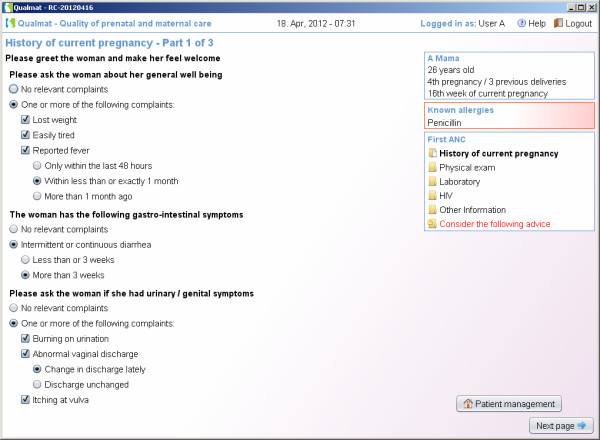
Decision support by electronic checklists: Guidance through routine actions in maternal and perinatal care is provided by checklists to ensure thorough clinical and laboratory work-up during antenatal care visits.

**Figure 4:**
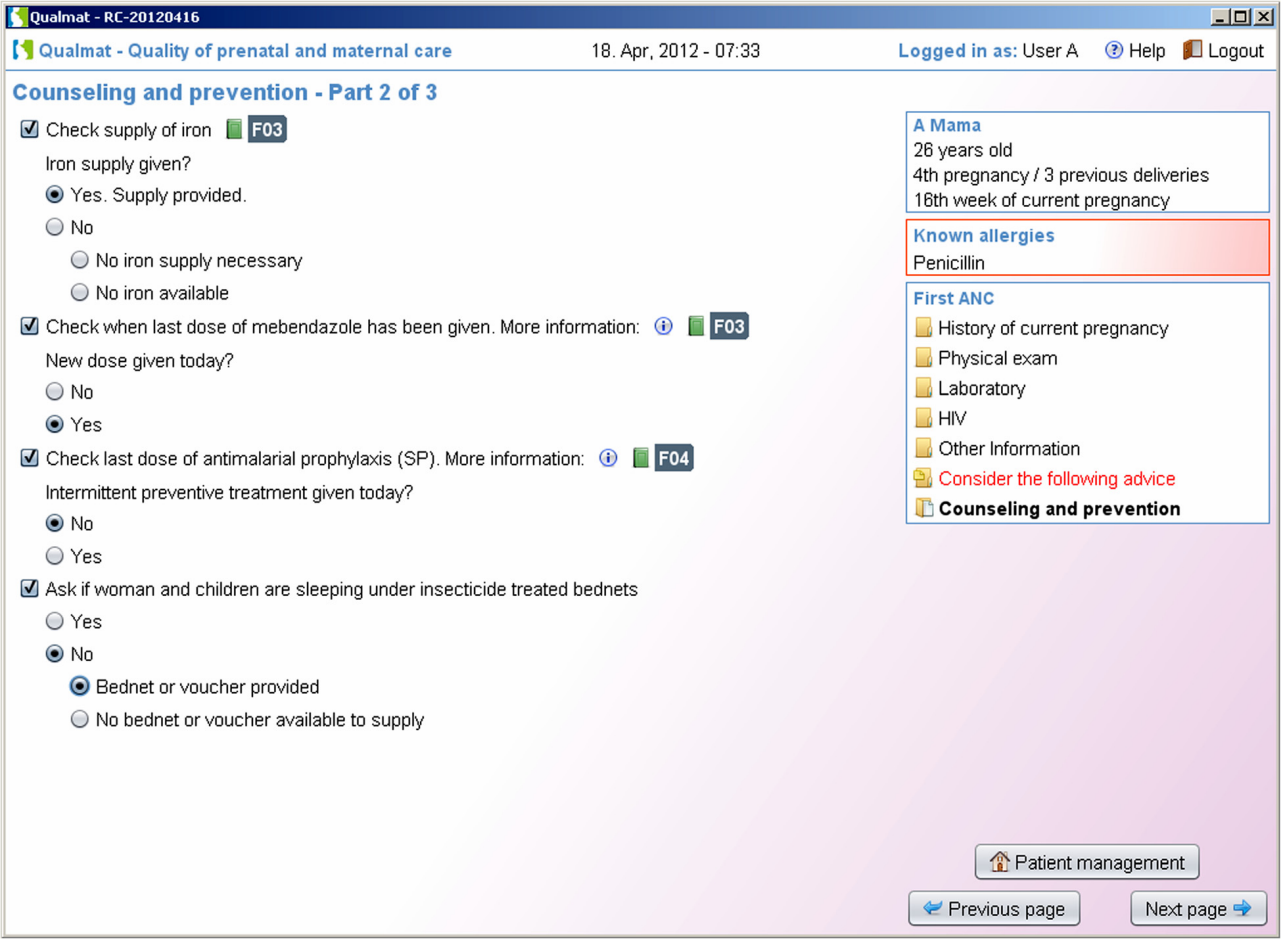
Decision support by electronic checklists: Guidance through routine actions in maternal and perinatal care: Checklists for preventive measures are provided.

The first approach is to provide decision support by electronic checklists, aimed to ensure that comprehensive information is available for decision making and all necessary actions for safe maternal care are observed and executed. This approach therefore supports thorough history-taking, physical examination, basic laboratory tests, as well as provision of counselling and preventive measures (Figures 3 and 4 give an examples). The results of these actions will be entered into the system and allow for review of all data from previous visits in the same way as in a patient’s chart.

**Figure 5:**
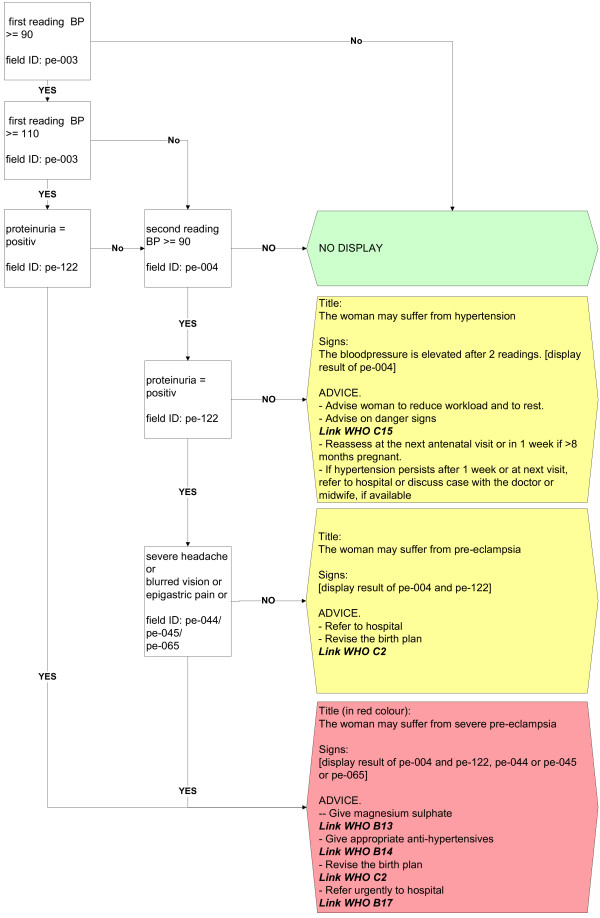
Algorithms for decision support by integration of data to detect situations of concern are derived from WHO guidelines.

**Figure 6:**
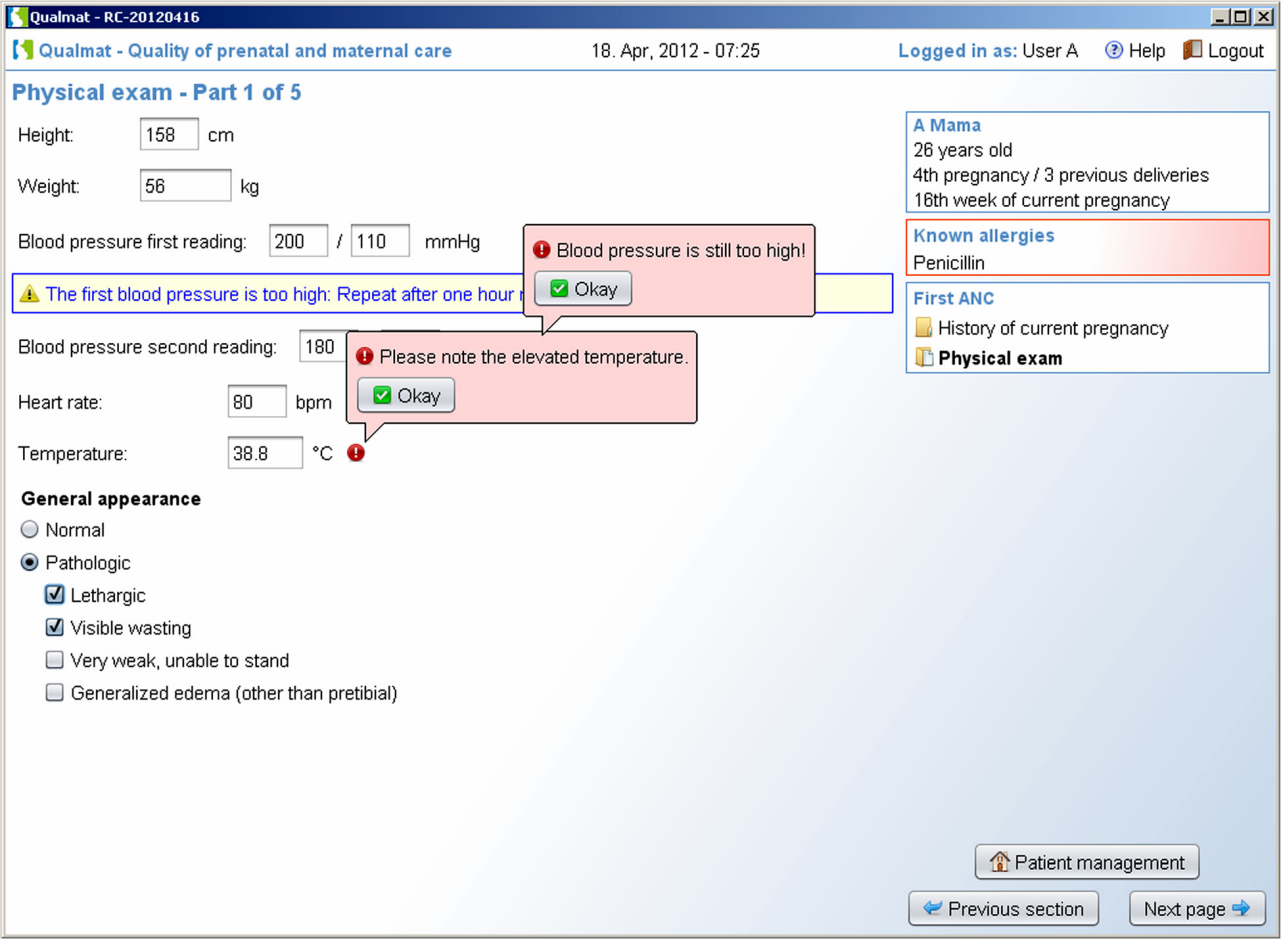
Algorithms detect situations of concern which result in immediate decision support through” watch dogs”.

**Figure 7:**
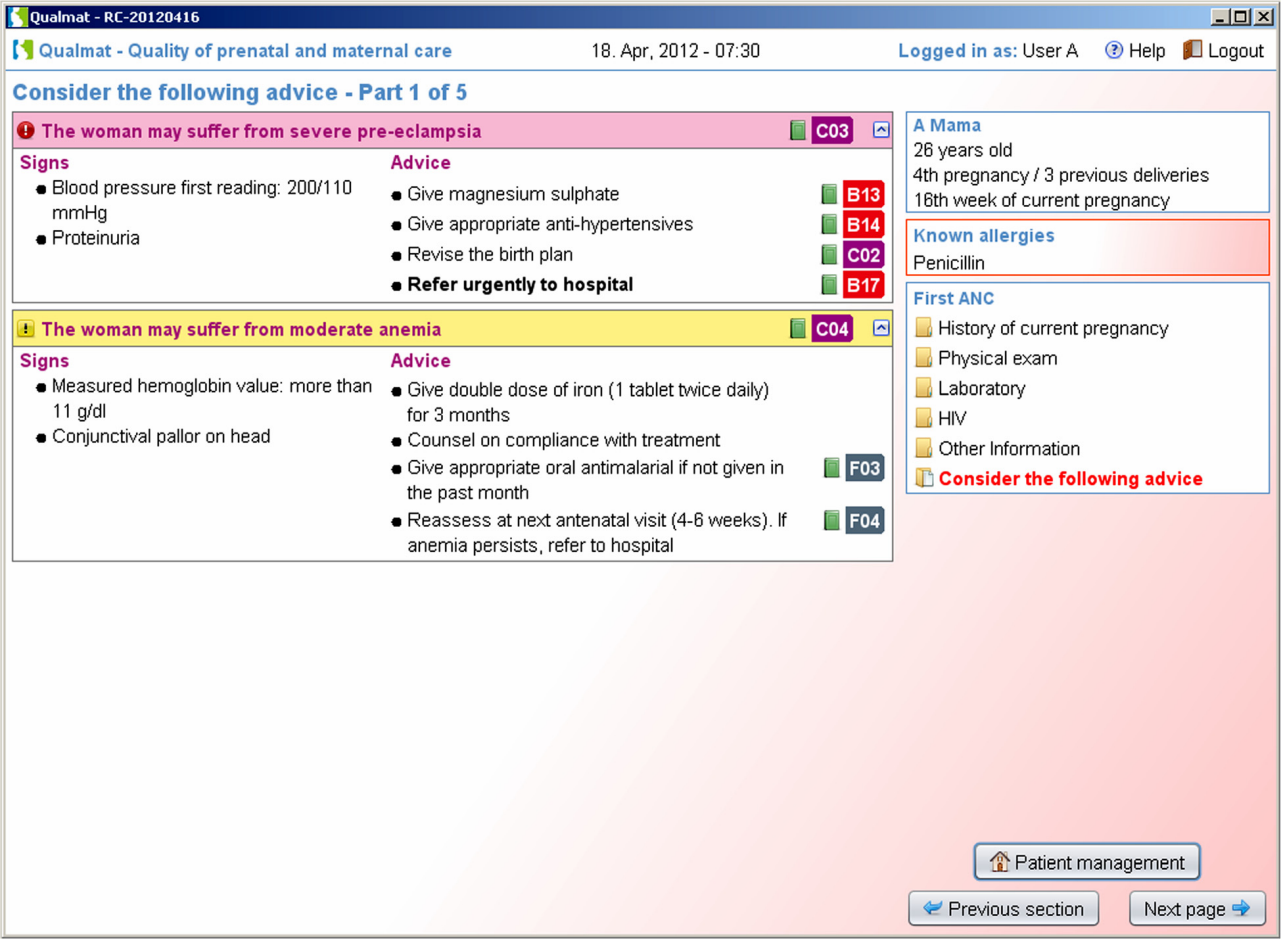
**Algorithms detect situations of concern which result in displaying detailed information including proposed diagnoses and actions.** A link for accessing source documents from WHO or local sources is provided (arrow).

The second approach is based on algorithms running in the background of the system that screen entered data and suggest diagnoses or alert the user about dangerous situations that require consideration during the visit (Figure 5). These algorithms consider information for example from the medical history, examination, basic laboratory test results. There are various options for displaying results of the analysis using the different algorithms. Algorithms may produce (i) different kinds of instant warnings (“watch dogs”, as shown in Figure 6), or they may (ii) integrate a broader amount of data to detect medical situations of concern and appear as more detailed hints at the end of a section, where diagnoses and actions (exams, treatment, or referral) are suggested. As an example, Figure 7 shows a situation, where the medical history revealed symptoms of blurred vision, the physical examination showed elevated blood pressure and the laboratory results showed proteinuria. At the end of the visit the caregiver was therefore asked to consider the diagnosis of preeclampsia. Within each recommendation the user is referred to the standard source (basic documents such as the WHO guideline itself or local guidelines if provided), which may be issued on demand (Figure 7, arrow).

**Figure 8:**
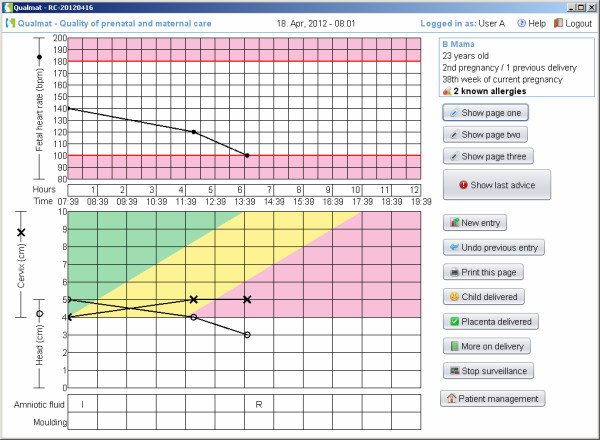
**Electronic partograph for visualisation of the progress of delivery.** The main screen with fetal heart rate and the degree of cervix dilatation is shown here as an example. In this example labour is delayed and the “alert line” of the partograph has been crossed.

**Figure 9:**
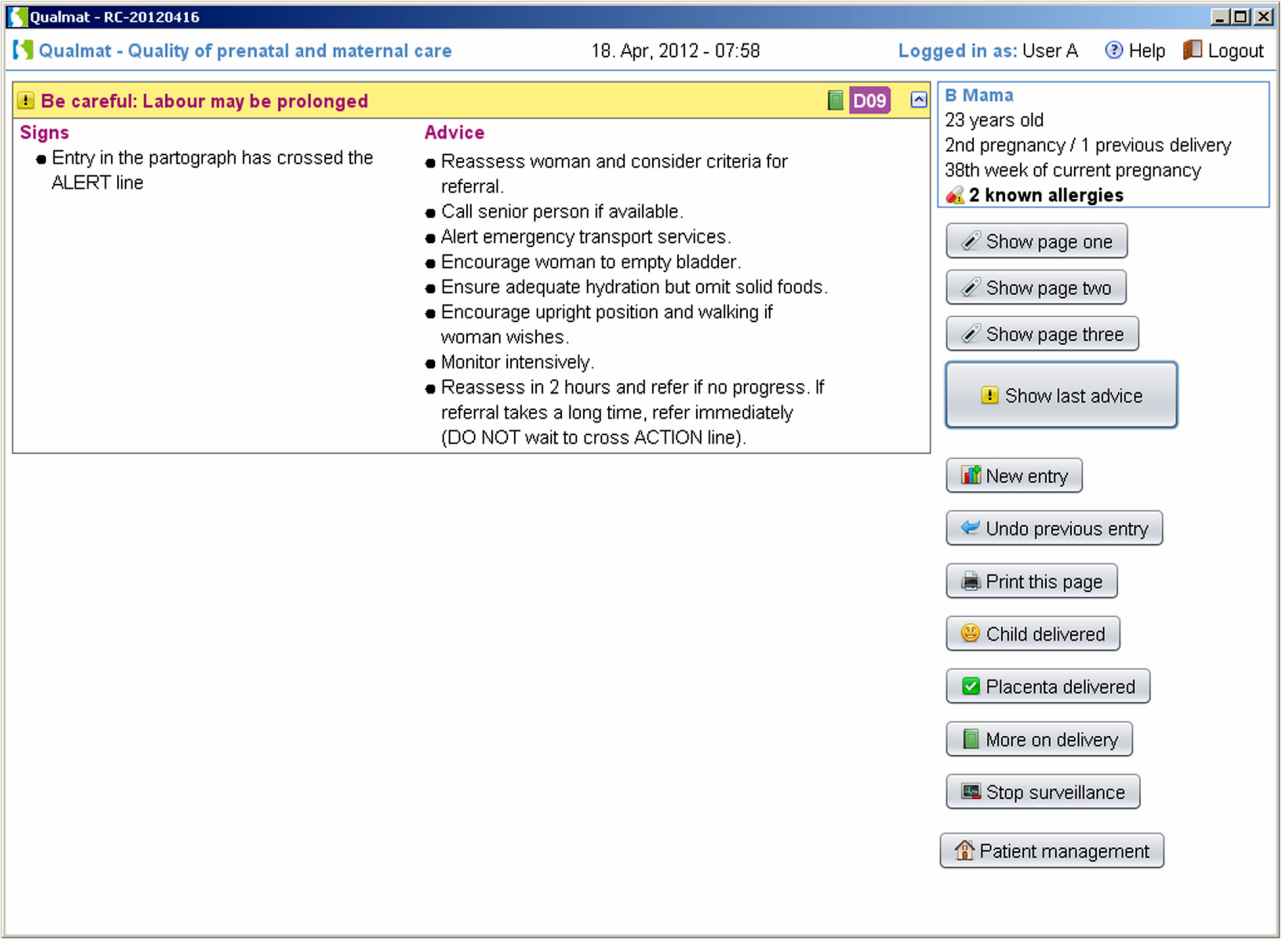
Electronic partograph: After crossing the alter line (entry at 4 h) the system will remind about the imminent risk and proposes action.

The third approach for decision support is the provision of an electronic partograph, which provides continuous monitoring of the delivery process on the screen and provides the above mentioned features of watch dog or detailed recommendations in addition. Figure 8 shows a partograph, where the entry for labour progress remained in the yellow alert area at 4 hours after the active phase of labour has started. At this time, the caregiver will receive detailed instructions (Figure 9). At 6 hours the action line has been crossed. Then there would be a red prompt which explains, that actions would be urgently necessary (Figure 9). A similar graph with decision support follows the postpartum phase until 24 h post delivery or until discharge.

#### Training section

**Figure 10:**
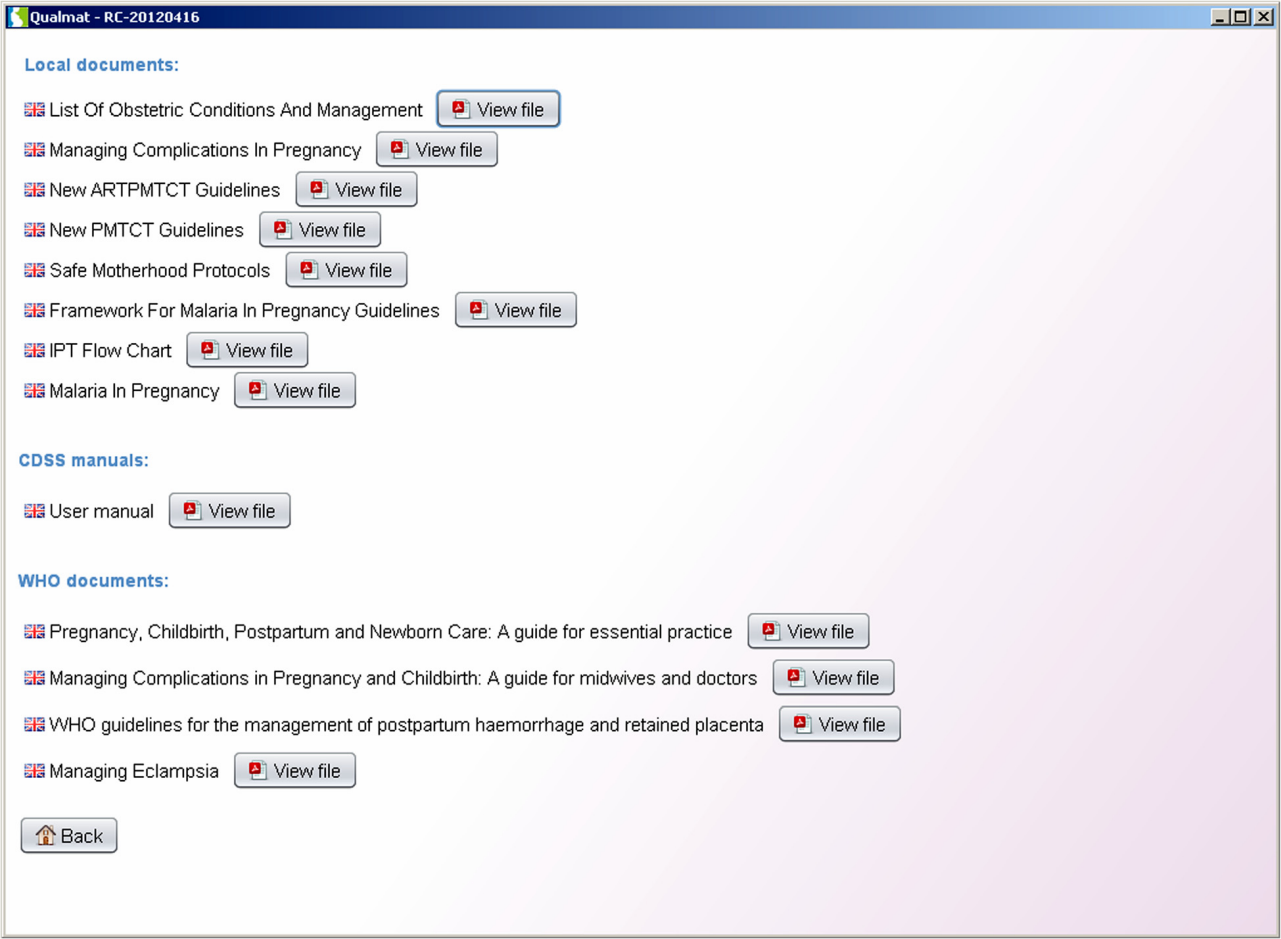
**Training section: Training documents are accessible without password for individual or group learning sessions.** Training documents can be added from different sources and on individual request (e.g. district medical officers).

The training section is offering documents for training and references for self reading. These documents are stored in a separate area of the system, which can be accessed without a personal password and can therefore be used by all site personnel. Key documents from WHO and national sources have been chosen by the local research teams and have been centrally uploaded, but local authorities, supervisors, or research staff may add additional documents to the system (Figure 10).

The features included into the software to support best medical practice and to promote usefulness and acceptability of the system are summarized in Table 1. This table lists critical factors for success of such systems in western countries as compiled by Kawamoto and colleagues [14]. Only few features could not be integrated; as an example redundant data entry could not be entirely avoided due to the fact that system use was restricted to participating health care facilities and patients were free to attend also other institutions not equipped with the QUALMAT software.

**Table 1:** **Features critical to success of electronic decision support **[14]** and corresponding characteristics of the QUALMAT CDSS development**

**Potential success factors**	**Corresponding features of the QUALMAT CDSS**
**General system features**	
Integration with charting or order entry system to support workflow integration	The system supports the workflow of ANC visits in guiding the nurse step by step through the process.
	The structure of the system is streamlined with the antenatal care cards which are the main documentation for pregnant women in these areas.
	The delivery process is followed dynamically, designed according to the present workflow.
Use of a computer to generate the decision	QUALMAT CDSS is computer based and will be operated from laptops at the health care facility.
	Women who are overdue for antenatal care can be identified automatically and contacted.
**Clinicial system interaction feature**	
Automatic provision of decision support as part of clinician workflow	Use of laptops dedicated to the QUALMAT software covers all regular and also unscheduled antenatal care visits, delivery, and the first 24h after delivery. Decision support is issued to the care provider for all relevant decisions during these visits.
No need for additional clinician data entry	This was not attempted because the ANC cards currently need to be completed in parallel (in case the woman delivers elsewhere). Printout of the cards is currently not implemented albeit possible as soon as printing infrastructure becomes available.
Request documentation of the reason for not following CDSS recommendations	If critical actions (e.g. laboratory tests, preventive measures) are not carried out the reason will be documented (e.g. patient’s refusal, missing equipment, other reason).
Provision of decision support at time and location of decision making	The mobile laptops can be used in any room of the health care center and decision support is therefore supported at the point of care.
Recommendations executed by noting agreement	Recommended actions during patient visits are marked as done or not done in the system. In addition, on a summary page at the end of each visit care providers are asked to indicate their agreement (or disagreement) with the proposed actions.
**Communication content features**	
Provision of a recommendation, not just an assessment	If situations of concern are detected the system provides detailed and explicit instructions on how to proceed.
Promotion of action rather than inaction	Specific actions are proposed such as referral of a patient, drug application, or interventions.
Justification of decision support via provision of reasoning	Recommendations can be instantly accessed through links to WHO and local guidelines integrated in the system.
Justification of decision support via provision of research evidence	Decision support is based on WHO and local guidelines. Because the provision of a scientific text appeared not to add benefit in a rural African environment, the provision of straightforward and user-friendly WHO guidelines was deemed appropriate.
**Auxiliary features**	
Local user involvement in development process	Local users were involved in each step of planning and during all test phases of the prototype and the release candidates.
Provision of decision support results to patients as well as providers	Patients will not directly see the results of the decision support, e.g. printouts, because the logistics in the given environment do not yet allow for this.
	However, providers are always able to review previous and current decisions suggested by the system.
CDSS accompanied by periodic performance feedback	CDSS is linked to a performance based incentives scheme. Users will receive regular performance feedback, where data from the CDDS are the major but not the only valuation source for the assessment.
CDSS accompanied by conventional education	Extensive training workshops have been held on general and specific computer use. During these workshops guidelines on maternal care were also part of the training schedule.

### Implementation

A CDSS for a resource-poor setting needs to strike a balance between the best technical possibilities and affordable, reliable, and robust technical solutions that can be used in rough rural environments. For the hardware environmental factors like high temperature, dust levels, and high humidity were taken into account; however the most suitable hardware, so called ruggedized laptops, was outside the tight research budget and may always be outside a health system’s budget in resource-poor countries. Therefore, affordable equipment was chosen that had been used previously in such settings and for which local maintenance could be ensured.

Study sites are equipped with one laptop per site with personal password protected accounts for each staff member involved in maternal care, as well as for supervisory staff. The infrastructure provides the possibility of having a laptop running 24 hours a day, seven days a week using mostly solar power and appropriate batteries. All staff received training to acquire general computer skills and specific knowledge required for safe handling of hard- and software and the collected data. As faulty hardware may be a powerful negative motivator and may thus interrupt local workflow and adversely affect patient care, round-the-clock technical support will be available provided by the local research team. A dedicated IT administrator visits each site regularly (at least bi-weekly) to identify and solve problems arising from soft- and hardware. This person is also responsible for the administration of local user accounts and passwords and for collecting data during the lifetime of the QUALMAT study. Having completed the appropriate training of future users in workshops, and after the iteration of test phases at the sites, the final version of the software has now been implemented and authorized for use in patient care at the intervention study sites in all three countries.

### Ongoing evaluation of the CDSS during implementation

The effect of the CDSS on the quality of maternal care, on the competence and motivation of the providers, and the cost-effectiveness of introducing such software will ultimately decide upon the success of the system. Therefore our project will closely monitor the introduction of the CDSS to facilitate timely recognition if problems of acceptance, hardware, software, or workflow hinder the implementation. Early adaptations, corrections and trouble-shooting as well as continuous discussions with and support of users may catalyze the adoption of the QUALMAT CDSS. This approach was successfully taken in a Kenyan project, where the early detection of problems through evaluation and the provision of corrective measures for problems helped to overcome the initial frustration of users [22]. Therefore a study program has already started to closely evaluate the adoption, usability as well as the attitudes towards the CDSS and its effects on the workflow at the different sites. Quantitative and qualitative assessments before, at certain time points during, and after the intervention will be carried out using questionnaires, interviews, observations and focus group discussions and will be supplemented with additional computer log analyses.

## Discussion

Over the years great attention has focused upon reinforcing health provider competence. Poor performance of health workers in resource-poor settings was attributed to insufficient knowledge and skills [10,23]. This resulted in a major investment in training, “which has had mixed and sometimes disappointing long-term results” [24]. Clinical guidelines and job aids such as flowcharts, checklists, algorithms, or a printed partograph were also frequently introduced to improve quality of care [25,26]. However, the effectiveness of disseminating paper-based work aids such as guidelines without additional interventions has been found lacking [24,27-30]. There are many possible reasons for this, including the guidelines’ availability at facility level, how they were introduced [31,32], or provider confidence to implement the proposed practice [27]. In a review undertaken within the QUALMAT study we found that poor accessibility of guidelines was common in the three countries involved [21]. Easy access to and easy application of guidelines is possible with the described CDSS-system, although it is recognised that this will only be achieved if implementation, maintenance of the system and user support – all interference prone issues – are carefully controlled.

In developed countries the implementation of guidelines has been notably enhanced by the use of HIT and computer systems [33-35]. In particular, the introduction of electronic decision support has been linked to improved physician performance, enhanced drug therapy safety through e.g. less medication errors. Decision support can help providers to apply knowledge more effectively and improve patient outcome if the systems are tailored to the needs of the users and acceptance is continuously monitored [13,14,36]. Resource-poor countries are also starting to experience the benefits of new technical options as HIT and mobile phone technology expand rapidly. Although there is sometimes reluctance to rely on electronic support in such environments, an increasing number of projects have shown promising results despite fragile infrastructure [37-40]. The majority of the projects focus on facilitating administrative tasks, whereas this project believes that supporting guideline-coherent decisions could have a greater impact on the quality of care in the targeted environment.

Indeed, the innovative aspect of QUALMAT is that HIT will be harnessed to support provider decision-making in rural, resource-poor settings. The decision support focuses on quality deficiencies across the continuum of antenatal care and delivery, all of which are target areas for reducing maternal deaths [41]. Studies have shown, for example, that routine actions are sometimes not executed (history taking, blood pressure measurement, preventive measures and counselling, or actions in third stage of delivery) [6,8,42] and diagnoses may not be recognized in time (e.g. pre-eclampsia or delayed birth progress). This may be due to a lack of knowledge, possibly based on insufficient training or low staff motivation resulting from a non-conducive working environment. The CDSS supports the execution of the complete actions necessary for antenatal care visits and delivery and helps to identify situations of concern, where timely actions like referral may reduce the likelihood of adverse consequences.

However, proposing tasks may not always result in actions as illustrated in the following three scenarios: (i) the software may not have detected a complex medical reality correctly resulting in the provider deciding not to follow an electronically proposed instruction. Such decisions must be respected, even encouraged and health workers using HIT should be aware that if their judgment and experience lead them to draw a different conclusion, then they are correct to overrule the decision support system; (ii) it may also be the case that a provider may not want to execute certain actions and may learn with time which information to withhold so as to avoid the system proposing them in the first place. This can lead to adulteration of data, which is difficult to detect. Situations where the electronically proposed actions are not carried out for various reasons including low provider motivation or poor work behaviour will be even more difficult to detect. And (iii) proposed actions may not be possible to be carried out due to missing equipment or infrastructure. This situation cannot be influenced by decision support, but it can be documented in the system and thus becomes evident. Decision support therefore provides supportive assistance rather than enforcement, which may be interpreted as weakness of the project’s approach, which is however inherent to most electronic systems. In anticipation of this weakness the QUALMAT software provides a range of relevant features that are supposed to enhance the usefulness and acceptance (Table 1). Indeed, ultimately only few features were not available. As an example, the handwritten antenatal card, as a central document for pregnant women in this setting, could not be replaced partly because printout of essential documents was not possible due to logistic reasons. The software, however, was designed accordingly and supports the printout of relevant documents.

The introduction of the CDSS was preceded by training and is now accompanied by long term local administrative support. It is expected that accessing computers, receiving computer training, and receiving regular follow-up with supervision will be appreciated by the providers, especially as health workers in rural areas report feeling “forgotten” [43,44]. Indeed, learning how to use the computer will likely contribute to the providers’ professional advancement. Furthermore, the CDSS may also diversify and enrich the task of delivering maternal care, reinforce provider confidence, and offer a way for health providers to improve their competence in maternal care.

In rural facilities, which suffer from shortage of staff and where health providers work in professional isolation, the introduction of such software will make the working environment more supportive. Finally, the rural first line health facilities equipped with the CDSS have gained access to enhanced power supply, with back-up possibilities, generators and solar panels through local purchasing channels. It is thus plausible that this variety of improvements to the work environment may jointly serve to enhance the motivation of the providers involved [16].

Implementing a CDSS in a resource-poor environment is undoubtedly challenging. The introduction of new software – in whatever context – can cause users to experience frustration due to software or hardware insufficiencies. After extensive and iterative discussion and testing of the software under development, after training, and after careful planning of local IT support, the taken measures will hopefully be sufficient to minimise user frustration and maximise the enthusiasm which has been witnessed so far.

## Conclusion

The QUALMAT CDSS is an intervention that is being introduced within the QUALMAT study. The implementation of a CDSS in a rural resource-poor setting is challenging. However, the difficulties can be mitigated if suitable measures are taken during the development phase. To increase the chances of success in such settings the CDSS takes a multi-pronged approach. It is anticipated that it will help to create an improved work environment resulting in enhanced provider motivation, support knowledge, and ensure stronger adherence to guidelines, all of which should finally influence the quality of maternal health care in a positive way.

## Abbreviations

CDSS: Clinical decision support system; GB: Gigabyte; HIT: Health care information technology; IT: Information technology; MMR: Maternal mortality ratio; QUALMAT: Quality of maternal care; WHO: World Health Organization.

## Competing interests

The authors declare not to have any competing interests.

## Authors’ contributions

WEH is scientific and project leader of the CDSS development, AB, JK, AK, WEH planned and developed the CDSS, AB and HP wrote the paper, FS, NM, AZ are responsible for the implementation of the CDSS in their country and contributed to the planning and development of the CDSS, HP is project leader of the motivational aspects of QUALMAT, SL is project manager of the QUALMAT consortium and contributed to planning of the CDSS, RS is the leader of the overarching QUALMAT project. All authors contributed to the paper. All authors read and approved the final manuscript.

## Pre-publication history

The pre-publication history for this paper can be accessed here:

http://www.biomedcentral.com/1472-6947/13/44/prepub
